# 

**Published:** 1895-12

**Authors:** 


### CONTENTS.

### COMMUNICATIONS.

Four Requisites in Development ........ 573
Report of Special Committee on Dental Nomenclature ........ 578
Nomenclature ........ 580
A Basis for Dental Nomenclature ........ 583
Whither are We Drifting? ........ 587
Should not the Increase of Dental Schools be Restricted ........ 588
Report of Section II ........ 589
American Dental Association ........ 590
Practical Notes ........ 593
Antiseptic Dental Surgery ........ 595
Resolutions Passed by the American Dental Association at the last Meeting ........ 596
The Æsthetic Correction of Facial Contours in the Practice of Dental Orthopedia ........ 597
Some Incompatibilities ........ 608
The Dentist in his Profession and Among the People ........ 611

### SELECTIONS.

A Purgative for Children ........ 589
Better Sutures ........ 615
Treatment of Cicatrices Pressure and Friction ........ 616
How Cocaine Kills ........ 617

### EDITORIAL.

Dentistry in Japan ........ 617

